# Quantification of Head Movement Predictability and Implications for Suppression of Vestibular Input during Locomotion

**DOI:** 10.3389/fncom.2017.00047

**Published:** 2017-06-07

**Authors:** Paul R. MacNeilage, Stefan Glasauer

**Affiliations:** ^1^German Center for Vertigo and Balance Disorders, University Hospital of MunichMunich, Germany; ^2^Center for Sensorimotor Research and Department of Neurology, Ludwig-Maximilian-University MunichMunich, Germany

**Keywords:** efference copy, motor, sensory, prediction, variability, natural statistics, angular velocity, linear acceleration

## Abstract

Achieved motor movement can be estimated using both sensory and motor signals. The value of motor signals for estimating movement should depend critically on the stereotypy or predictability of the resulting actions. As predictability increases, motor signals become more reliable indicators of achieved movement, so weight attributed to sensory signals should decrease accordingly. Here we describe a method to quantify this predictability for head movement during human locomotion by measuring head motion with an inertial measurement unit (IMU), and calculating the variance explained by the mean movement over one stride, i.e., a metric similar to the coefficient of determination. Predictability exhibits differences across activities, being most predictable during running, and changes over the course of a stride, being least predictable around the time of heel-strike and toe-off. In addition to quantifying predictability, we relate this metric to sensory-motor weighting via a statistically optimal model based on two key assumptions: (1) average head movement provides a conservative estimate of the efference copy prediction, and (2) noise on sensory signals scales with signal magnitude. The model suggests that differences in predictability should lead to changes in the weight attributed to vestibular sensory signals for estimating head movement. In agreement with the model, prior research reports that vestibular perturbations have greatest impact at the time points and during activities where high vestibular weight is predicted. Thus, we propose a unified explanation for time-and activity-dependent modulation of vestibular effects that was lacking previously. Furthermore, the proposed predictability metric constitutes a convenient general method for quantifying any kind of kinematic variability. The probabilistic model is also general; it applies to any situation in which achieved movement is estimated from both motor signals and zero-mean sensory signals with signal-dependent noise.

## Introduction

During everyday actions, the brain can estimate achieved movement either based on sensory signals that indicate how the body has moved or based on predictions derived from efference copies of motor commands. The degree to which the brain relies on the sensory vs. the motor estimate is thought to depend on the noise associated with these estimates, with less noisy estimates getting greater weight. This weighting scheme is well-described by the Maximum-likelihood (ML) model of cue integration (van Beers et al., 1999; Ernst and Banks, 2002).

Testing the model requires methods to quantify sensory and motor noise. Traditional methods rely on 2-interval-forced-choice tasks for quantifying sensory noise (Ernst and Banks, 2002), or analysis of trajectory endpoints for quantifying motor (and/or proprioceptive) noise^1^ (van Beers et al., 1999). However, alternative methods are required for continuous motor behaviors such as locomotion, which is the focus of the current study. Here we suggest that sensory and motor noises can be roughly approximated based on continuous kinematic measurements. In particular, we record 6-degree-of-freedom head motion during walking and running using an inertial measurement unit (IMU). We first describe a method for quantifying kinematic variability and predictability based on these measurements. We then continue to develop a model to relate these measures to sensory and motor noise and weight during locomotion.

Recordings for a given participant are first divided into strides and then averaged to reconstruct the mean head motion over one stride. Total variability is calculated by squaring and summing this average trace. Residual variability is calculated by squaring and summing the amount by which each motion sample deviates from the average. Finally, predictability or stereotypy of head motion is quantified using the well-known coefficient of determination (i.e., R^2^ statistic), which is the ratio of residual to total variability. We refer to this quantity as the kinematic predictability metric (KPM) and propose it as a straightforward alternative to previously published measures of kinematic predictability or stereotypy.

To relate these measures to sensory and motor noise, we make several assumptions. We assume that an efference copy of stepping behavior is used to increase the accuracy of the head motion estimate. We do not have direct access to the efference copy signal for each step so we estimate it as the average head motion over all strides. Deviation from this average head motion on each stride is due to a combination of (1) intended deviation, (2) external perturbation, and (3) motor noise. If we assume that intended deviation and external perturbation are small, the residual variability measure described above provides an estimate of motor noise^2^. Even if the above assumption is violated, the approximation is still useful; residual variability can alternatively be interpreted as an upper limit on motor noise, an idea we return to in the discussion.

To estimate sensory noise from kinematic measurements we rely on the often-observed phenomenon that noise scales with the magnitude of the signal, i.e., that noise is signal-dependent. In this case, sensory noise should be proportional to the total variability measure described above, calculated as the sum of squares of the average trace. This approximation is useful even if the sensory signal is derived from multiple sensory modalities (e.g., visual, vestibular, proprioceptive), a point we return to in the discussion.

To derive sensory and motor weights, we substitute these noise values into the equations that describe sensory-motor weighting under the ML integration model. The equations express weight as dependent on the ratio of motor-to-sensory noise, which we approximate as residual-to-total variability. Thus, the KPM, calculated solely based on kinematic measurements, can be used to generate a prediction of the ideal sensory-motor weighting for any behavior that satisfies the model assumptions.

We use the model to predict how vestibular sensory weight should change during locomotion. Resulting predictions correspond well with previously observed changes in vestibular weight, both over time within a given stride, as well as across different locomotor activities. In particular, it has been shown that vestibular perturbation applied via Galvanic Vestibular Stimulation (GVS) leads to significant perturbations in subsequent foot placement only when the stimulus is applied at a particular time during the stride cycle, e.g., at the time of the heel strike (Bent et al., 2004). Similarly, stochastic vestibular stimulation (SVS) leads to highly correlated EMG activity in the locomotor muscles of the leg during the same period of the stride cycle (Dakin et al., 2013). Our analysis of vestibular and motor noise based on measurement of head motion suggests that vestibular weight should indeed be highest at these time points.

Furthermore, the present model and measurements can also account for differences in vestibular weighting observed across activities. In particular, susceptibility to vestibular perturbations has been shown to be greater during walking than during running in both healthy subjects and vestibular patients (Brandt et al., 1999; Jahn et al., 2000). This matches with our observation that head movement is more predictable during running than during walking.

In addition, these findings match physiological observations of modulation of vestibular responsiveness during active movement across a range of animal studies. For example, studies in tadpoles have shown that vestibular signals are suppressed and replaced by locomotor efference copies during active swimming (Lambert et al., 2012). Similarly, vestibular signals in the vestibular nucleus and cerebellum are attenuated during active head movement in monkeys (Roy and Cullen, 2004).

## Methods

### Subjects

Ten subjects (5M, 5F) aged 20–37 participated in this study. Height ranged from 163 to 193 cm and weight ranged from 47 to 84 kg. Informed written consent was obtained prior to participation and all procedures were approved by the ethics committee of the LMU medical faculty.

### Procedure

All subjects did the activities for 6 min each, one after the other with breaks in between during one long session in the same order: running, walking in the field, walking on pavement, walking stairs. During walking stairs, subjects spent approximately the first 3 min. walking up stairs and the remaining time walking back down. An IMU (MTx sensor from Xsens) was strapped to the left side of the head with an elastic headband. The sensor transmitted 3-degree-of-freedom (DOF) measurements of linear acceleration, angular velocity, as well as orientation at 150 Hz sampling rate. A wire ran from the sensor to a lightweight laptop carried by the subject in a backpack. Post-hoc analysis confirmed that the sensor remained securely strapped to the head, except for one subject during walking on pavement where it was evident that sensor orientation relative to the head changed during the course of recording, so data for this subject and activity had to be excluded from further analysis.

Calibration was necessary to measure and correct for the misalignment of the sensor from Reid's plane. This calibration was performed before each activity. During calibration the subject stood with the back of the head against a flat surface and the experimenter used a level to align the pitch of the head such that Reid's line (defined as the line passing through the canthus of the eye and the meatus of the ear) was perpendicular to gravity. Subjects were otherwise instructed to hold the head upright, thus it was assumed that the head roll relative to gravity was zero. Once the proper orientation was achieved, the subject remained in that position for 60 s. The mean direction of the acceleration vector over this period is taken to be the normal to Reid's plane, and all data is transformed such that the direction of the z-axis is aligned with this vector.

### Stride identification and exclusion

Head motion data for each activity and subject was divided into individual strides, defined as the time between two consecutive footfalls with the same foot, as in previous work (e.g., Hirasaki et al., 1999; Mulavara and Bloomberg, 2002). Footfalls were identified by the instants of maximum z-axis linear acceleration. In experiments that have recorded both foot pressure and head acceleration simultaneously it has been observed that peak z-axis acceleration occurs during mid-stance, which is after heel-strike and before toe-off (Mulavara and Bloomberg, 2002).

Our analysis depends on identifying the average head motion across the stride for each subject and activity to find what we call the stride-cycle attractor, and we chose to normalize stride duration before averaging. Stride data was resampled to a total of 200 samples per stride via spline interpolation (“interp1” function in Matlab). Thereafter, sample number, or equivalently proportion of elapsed stride, was used as the index of time within the stride. The average stride duration for each activity was as follows: RUN (running) 0.75 (±0.02) s, WAP (walking on pavement) 1.10 (±0.06) s, WAF (walking on field) 1.13 (±0.06) s, WUP (walking up stairs) 1.24 (±0.11) s, WDN (walking down stairs) 0.90 (±0.14) s.

Because our focus is on head motion during steady-state locomotion, we excluded irregular strides (caused for example by events such as tripping, stopping, or tying a shoelace) as follows. Half-strides (i.e., inter-footfall intervals) were identified as outliers if their duration was too long or too short [i.e., >Q3+1.5(IQR) or < Q1 - 1.5(IQR)]. The outliers were excluded and strides were extracted following such outliers only if there were 20 consecutive acceptable half-strides (10 full strides) immediately following the outlier. If this criterion was not met, all data between consecutive outliers was discarded. This resulted in discarding the following percentage of the data, on average (SD), across subjects: RUN 1 (±1)%, WAP 3 (±3)%, WAF 3 (±3)%, WUP 7 (±5)%, and WDN 11 (±11)%. The average number of strides collected per subject for each activity was as follows: RUN 477 (±15), WAP 311 (±23), WAF 299 (±38), WUP 144 (±34), and WDN 140 (±49).

### Quantifying predictability

Predictability or stereotypy of head motion is quantified based on measured variability in head motion. In particular, we calculate the total head motion variability (*SS*_*tot*_) and quantify the proportion which can be predicted or explained by the stride-cycle attractor (*V*_*exp*_, also known as the coefficient of determination or R^2^) and the proportion of residual or unexplained variance (*V*_*res*_) across a total of N strides as follows,

(1) $$V_{exp}\;+\;V_{res}=1$$

(2) $$V{\left(t\right)}_{res}=SS{\left(t\right)}_{res}/SS{\left(t\right)}_{tot}$$

(3) $$SS{\left(t\right)}_{tot}=\frac{1}{N}\sum_{i=1}^{N}\sum_{d}{\left(m{\left(t\right)}_{d,i}\;-\;{\bar{m}}_{d}\right)}^{2}$$

(4) $$SS{\left(t\right)}_{res}=\frac{1}{N}\sum_{i=1}^{N}\sum_{d}{\left(m{\left(t\right)}_{d,i}\;-\;f{\left(t\right)}_{d}\right)}^{2}$$

${\bar{m}}_{d}$ is average head motion along dimension *d* (either x, y, or z axis) over all strides and times. *m(t)* is head motion measured at normalized stride time *t*, on stride number *i* along dimension *d. f(t)* is the average head motion for that stride time and dimension (i.e., the corresponding value from the stride-cycle attractor). Thus, *SS*_*res*_ quantifies signal deviation from the stride-cycle attractor, and *SS*_*tot*_ quantifies deviation from the mean signal. Note that deviations are expressed as Euclidean distances, which allows summing across dimensions to obtain a single metric of multidimensional variability.

### Relating predictability to sensorimotor weighting

In accordance with the widely accepted MLE model of cue integration (e.g., van Beers et al., 1999; Ernst and Banks, 2002), we assume that head motion (H) is estimated as a weighted linear combination of vestibular (sensory, S) and efference copy (motor M) signals with weights (w) determined by the relative reliability (or variability) of these signals:

(5) $$\hat{H}=w_{sens}\;\cdot \;S\;+\;w_{mot}\;\cdot \;M$$

(6) $$w_{sens}=\frac{\sigma_{mot}^{2}}{\sigma_{sens}^{2}\;+\;\sigma_{mot}^{2}}\;w_{mot}=\frac{\sigma_{sens}^{2}}{\sigma_{sens}^{2}\;+\;\sigma_{mot}^{2}}$$

These weights can be calculated based on the head movement measurement data given the following assumptions. First, we assume that sensory noise is signal dependent, i.e., that its variance is proportional to the squared signal. If our signal has zero mean (i.e., ${\bar{m}}_{d}=0$), which is approximately true for these oscillatory motions after subtracting gravity, we can then express sensory noise relative to our head movement measurements as $\sigma_{sens}^{2}=k\cdot {SS}_{tot}$ where *k* < 1 is an unknown proportionality constant. We assume a single multimodal sensory signal coding head motion in space that is determined predominantly by vestibular input but can include other components such as optic flow, proprioceptive, or somatosensory signals. We focus on vestibular signals here because we attempt to explain observations of changes in vestibular weight reported in the literature. If contributions of other sensory modalities are large, vestibular weight will be reduced accordingly. Finally, we assume that the intended/expected head movement on every stride is equal to the stride cycle attractor. Therefore, efference copy/motor noise is equal to the deviation of actual head movements relative to this attractor, which can be expressed as $\sigma_{mot}^{2}={SS}_{res}$. Substituting and rearranging, we now predict that sensory weight should depend on the proportion of residual variance of head motion relative to the stride cycle attractor as follows

(7) $$w_{sens}=\frac{SS_{res}}{k\;\cdot \;SS_{tot}\;+\;SS_{res}}=\frac{V_{res}}{V_{res}\;+\;k}$$

Notice that this model of ML sensory-motor weighting generalizes to any situation in which achieved movement is estimated from both efference copy and a zero-mean sensory signal with signal-dependent noise; weight given to the sensory signal decreases for more predictable, repeatable, and/or stereotyped movements.

Interestingly, Equation 7 implies an upper limit for the sensory weight given that the stride cycle attractor is not degenerate (i.e., not a constant): in this case *V*_*res*_ ≤ 1, because the residual variability *SS*_*res*_ will always be smaller than the total variability *SS*_*tot*_. Therefore, *w*_*sens*_ ≤ 1/(1 + *k*) < 1, which means that the contribution of sensory signals to the estimate of achieved movement cannot exceed an upper bound which depends on *k*, the proportionality constant for signal dependent noise. The estimate will *always* be determined in part by the efference copy.

## Results

**Figure 1:**
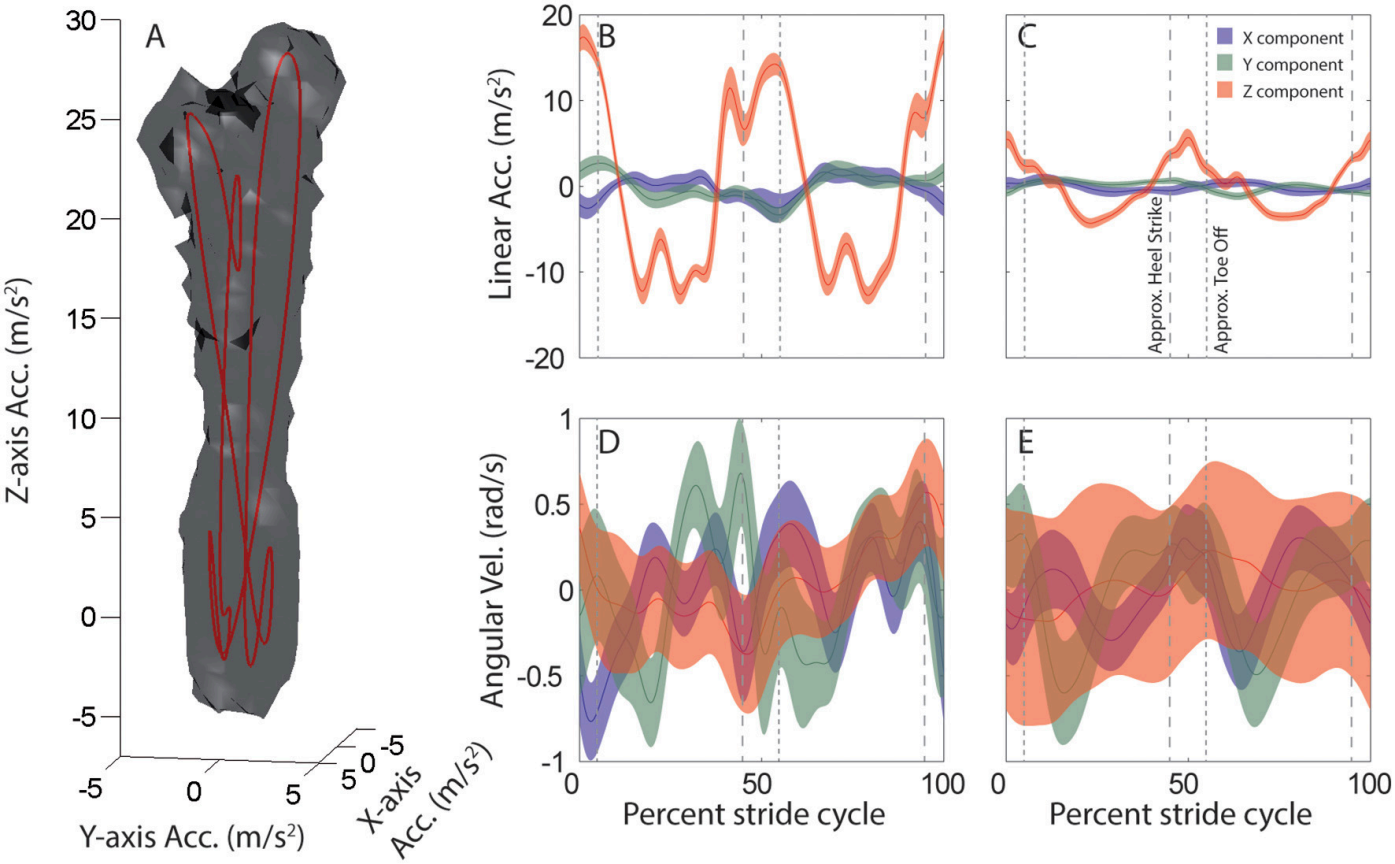
Average head motion during locomotion: example subject. **(A)** Stride-cycle attractor for linear head acceleration during running. Linear acceleration samples over the 6 min. recording period form a 3D point cloud in the space of possible head accelerations. The surface of this cloud is rendered at a density of 20 points per cubic cm/s^2^. Mean acceleration over the stride cycle defines an attractor in this space (red line). **(B–E)** Stride-cycle attractors for linear (top) and angular (bottom) head motion during running (left), and walking on a field (right) for an example subject. Traces illustrate the x (blue), y (green), and z (red) components of head motion averaged over all strides. Shaded area indicates SD across all strides. Vertical dashed lines are approximate timepoints of heel strike and toe-off, computed as 5% stride-cycle timing before and after point of maximum z-axis acceleration. Note, linear acceleration plotted in the upper panels is only the component of acceleration due to linear head motion, i.e., the total acceleration (as in A) minus the gravitational component. Orientation data from the sensor was used to estimate and then subtract the gravitational component at each time point.

Linear and angular head motion across the 6-min recording period can be visualized for each subject and activity as distinctive 3D point clouds (e.g., Figure 1A). Due to the periodic nature of the head motion it is possible to identify an attractor in each point cloud, which is calculated as the average linear (Figures 1B,C) and angular (Figures 1D,E) head motion during one stride.

**Figure 2:**
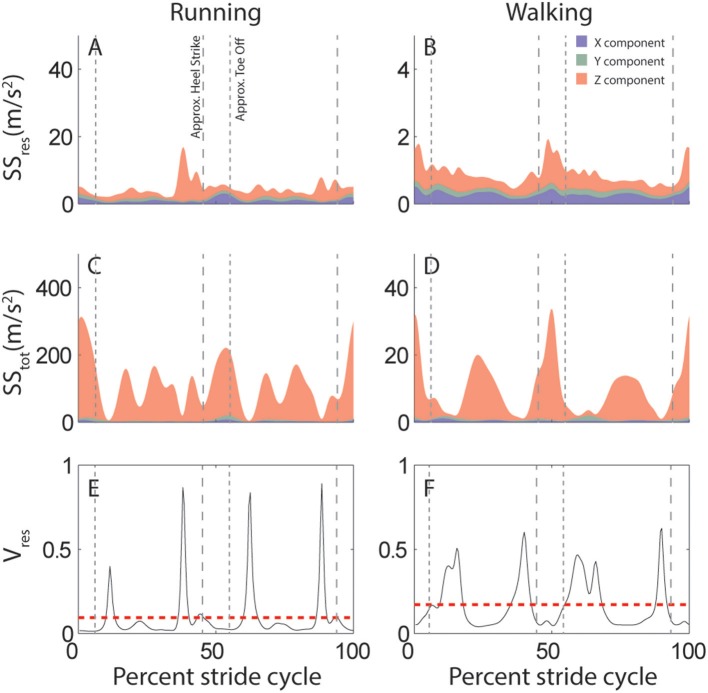
Variability of linear acceleration during running and walking: example subject. **(A,B)** Variation in head motion relative to the stride-cycle attractor (*SS*_*res*_) calculated as the sum of squared residuals (Equation 4). Shown is the mean *SS*_*res*_ across all strides for this subject for running **(A)** and walking **(B)**. Colors indicate the separate x (blue), y (green), and z (red) components of variability. Note y-axis scale is an order of magnitude larger for running than walking. **(C,D)** Total variation in head motion (*SS*_*tot*_) calculated as the sum of squared deviation from the signal mean (Equation 1). Shown is the mean *SS*_*tot*_ across all strides for this subject for running **(C)** and walking **(D)**. Note y-axis scale is an order of magnitude larger for *SS*_*tot*_ **(C,D)** than *SS*_*res*_ **(A,B)**. **(E,F)** Proportion of residual variance (*V*_*res*_, black line) calculated as the ratio of residual to total variance (Equation 2) for each time point in the stride-cycle for running **(E)** and walking **(F)**. The model predicts that vestibular signals should be weighted most highly during time points in the stride with a high proportion of residual variance. The dashed red line shows the mean *V*_*res*_ across the mean stride which provides a single value to quantify either linear or angular head motion variability for each subject and activity. Note that even though overall variance is greater during running **(A,C)** than walking (**B,D**;see y-axis scales), proportion of residual variance (*V*_*res*_) is reduced (E<F). Vertical dashed lines are approximate timepoints of heel strike and toe-off, computed as 5% stride-cycle timing before and after point of maximum z-axis acceleration.

This attractor is further used to quantify the predictability of head motion. Specifically, for each subject and activity we calculate the proportion of variance that cannot be explained by the stride-cycle attractor, i.e., the proportion of residual variance (*V*_*res*_, Equations 1, 2). This quantity depends on total variance (*SS*_*tot*_), which is calculated as deviation from the mean signal (Equation 3), and residual variance (*SS*_*res*_), which is deviation from the stride-cycle attractor (Equation 4). The approach is illustrated in Figure 2 which compares variability of linear head motion during running (left) and walking (right) for the same individual subject shown in Figure 1. The top row (Figures 2A,B) shows residual variance (*SS*_*res*_), the middle row (Figures 2C,D) shows total variance (*SS*_*tot*_), and the bottom row (Figures 2E,F) plots the ratio of the two, which is the proportion of residual variance (*V*_*res*_). A value of *V*_*res*_ closer to zero means that head motion can be predicted very well based only on knowledge of where you are in the stride cycle, information that is readily available from central pattern generator or efference copy signals during locomotion. On the other hand, a higher value of *V*_*res*_ indicates less predictive value of stride-cycle timing information and thus increased expected utility/importance of vestibular sensory signals for estimating head motion. This intuition is expressed mathematically in Equation 7, where larger values of *V*_*res*_ correspond to larger sensory weights.

**Figure 3:**
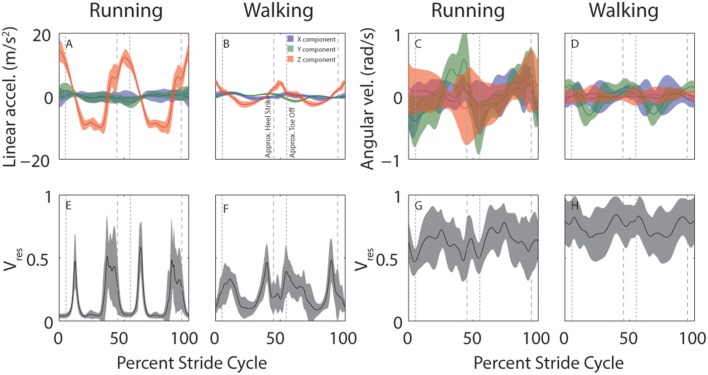
Across-subject average head motion (top) and residual variance (bottom) during running and walking. **(A–D)** Average stride cycle for linear **(A,B)** and angular **(C,D)** head motion during running **(A,C)** and walking **(B,D)**. Colors indicate the separate x (blue), y (green), and z (red) components. **(E–H)** Average proportion of residual variance (*V*_*res*_) for linear **(E,F)** and angular **(G,H)** head motion during for running **(E,G)** and walking **(F,H)**. Shaded area indicates ±SD across subjects. Vertical dashed lines are approximate timepoints of heel strike and toe-off, computed as 5% stride-cycle timing before and after point of maximum *z*-axis acceleration.

Despite considerable across-subject differences in height, weight, and gender, there are commonalities in how head motion and head motion variability are modulated across the stride cycle. This is illustrated in Figure 3 which shows across-subject mean and SD for linear (left) and angular (right) head motion (top row) and *V*_*res*_ (bottom row) for both running and walking. Variability across subjects appears greater for angular than linear head motion. This is likely because the range of angular head motion is rather small, so even minimal differences in angular head motion across subjects results in standard deviations that appear large in comparison.

It is interesting to note the distinctive peaks in residual variance (*V*_*res*_) for linear head motion (Figures 3E,F). These peaks indicate time-points during which head motion is not well-predicted, such that vestibular signals might be expected to play a more critical role. These peaks occur just before and just after the peaks in z-axis linear head acceleration (Figures 3A,B, red). Maximum z-axis acceleration occurs during mid-stance (Mulavara and Bloomberg, 2002), so the peaks in *V*_*res*_ correspond roughly to heel-contact and toe-off which occur before and after maximum z-axis acceleration, approximately when z-axis acceleration is near zero. Studies that have examined the phase dependence of vestibular influence on locomotion have identified exactly these time points as the ones where vestibular influence is maximal (Bent et al., 2004; Dakin et al., 2013).

**Figure 4:**
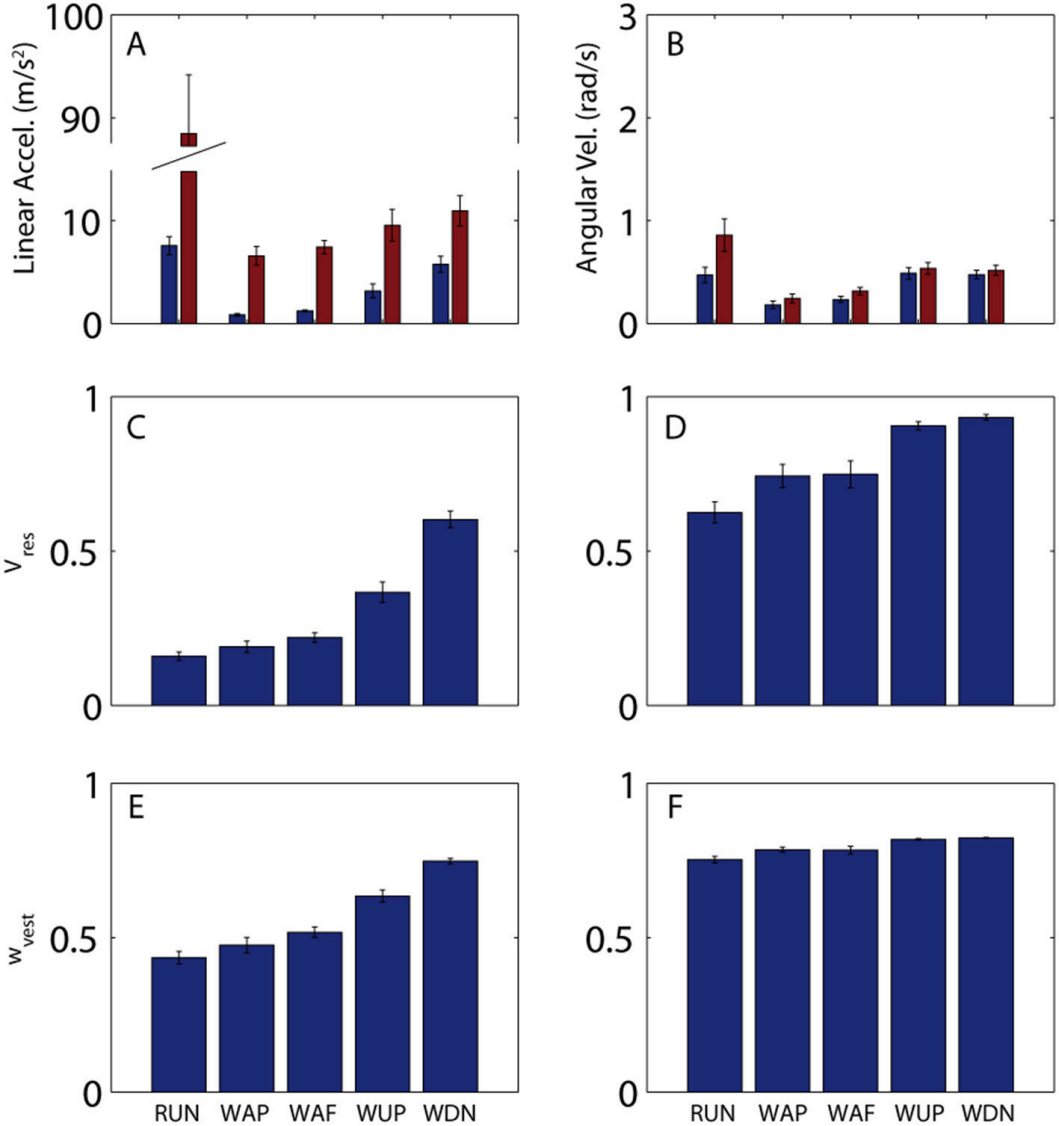
Variability of head motion and predicted vestibular weight across subjects and activities. **(A,B)** Residual (blue) and total (red) variability for linear acceleration **(A)** and angular velocity **(B)** during running (RUN), walking on pavement (WAP), walking on a field (WAF), walking up stairs (WUP), and walking down stairs (WDN). A single value was calculated for each subject and activity as the mean across the mean stride (e.g., mean values from Figures 2A–D). Shown here are the mean (±SE) values across the 10 subjects. **(C,D)** Proportion of residual variance for linear acceleration **(C)** and angular velocity **(D)** for all activities. A single value was calculated for each subject and activity as the mean across the mean stride (e.g., mean values from Figures 2E,F). Shown here are the mean (±SE) values across the 10 subjects. **(E,F)** Predicted sensory (vestibular) weight for each activity calculated according to Equation 7 using the *V*_*res*_ values shown in **(C,D)** and a hypothetical value of *k* = 0.2 as the proportionality constant for signal-dependent noise. Shown here are the mean (±SE) values across the 10 subjects. Note that while *V*_*res*_ can reach a value of 1, sensory weight cannot exceed a maximum value equal to 1/(1 + k); this limit is approached in **(F)** (see text for additional discussion on this point).

In addition to large variations in predictability across the stride for a given activity, there are considerable differences across activities. Figure 4 shows across-subject averages of total and residual variability (Figures 4A,B) for all activities, as well as *V*_*res*_ (Figures 4C,D), and hypothetical vestibular weight (Figures 4E,F). Proportion of residual variance (*V*_*res*_) was minimal for running (RUN), meaning that head motion was well-predicted by the stride-cycle attractor. *V*_*res*_ increased for walking on pavement (WAP) and field (WAF) and was greatest for walking up (WUP) and down (WDN) stairs. The change in proportion of residual variance across activities was not driven solely by residual variance (*SS*_*res*_, numerator) or total variance (*SS*_*tot*_ denominator), both of which showed a different pattern of modulation across activities (Figure 4, top). Most notably for running, total variance is approximately one order of magnitude greater than for other activities, while residual variance remains of the same order as for the other activities, which leads to the lowest *V*_*res*_ for this activity. In fact, head motion is significantly more predictable during running compared to walking on a field (paired *t*-test on *V*_*res*_ values; linear, *p* < 0.001; angular, *p* = 0.07). This seemingly paradoxical result can explain why patients with vestibular deficits and healthy participants exposed to vestibular perturbations are less susceptible to adverse vestibular activation during running than during walking (Brandt et al., 1999; Jahn et al., 2000).

In addition to explaining vestibular reliance, head motion predictability provides a convenient metric for quantifying differences in stability, even within a given activity. For example, *V*_*res*_ is less for walking on pavement than for walking on a field (linear, *p* = 0.04; angular, *p* = 0.4), and less for ascending than for descending stairs (linear, *p* < 0.001; angular, *p* = 0.22), observations that also match with qualitative differences in postural/locomotor stability across these activities. In both these cases, differences in *V*_*res*_ are driven primarily by differences in residual rather than total variance (Figure 2, top). Thus, while both total and residual variance can vary considerably across activities, it is ultimately the ratio of the two that provides the best quantification of postural stability and vestibular reliance.

Even though predictability of linear and angular head motion co-vary across activities (rho = 0.69, *p* < 0.001), linear head motion is much more predictable than angular (*p* < 0.001). This suggests that efference copy-based mechanisms will be more useful for linear than angular stabilization. Conversely, angular stabilization should be more dependent on vestibular signals. A related prediction is that patients with localized damage to the canals should be more impaired than those with only damage to the otoliths.

## Discussion

Above we proposed a KPM based on the coefficient of determination for the average, time-normalized trajectory over repeated movements (Equations 1–4) and we applied this method to head motion recordings during various locomotor activities. With some assumptions, we show that this measure should reflect the relative weight given to sensory and motor signals for estimating head movement (Equations 5–7). Below we discuss these assumptions and we show that predictions of the model are consistent with previously observed differences in vestibular weight across activities, as well as over time within a single stride. We also show that the proposed vestibulo-motor interactions are consistent with known physiological convergence and vestibular suppression in animals. While these relationships are suggestive, they do not prove the model. We therefore discuss possible experiments that would test the model. We conclude with speculation about clinical applications of the KPM and generalizability of the sensorimotor weighting model.

### Assumptions for deriving sensory and motor noise from kinematic data

To derive motor noise, we first calculate the mean head motion during one stride cycle and we assume that this is equivalent to the intended head motion on every stride. However, the efference copy during an individual stride can be supposed to carry much more information than just the average amplitude over all strides. If, for example, a subject encounters an obstacle which requires them to alter their stereotypical stepping pattern, or if they simply turn their head to look at something eccentric to their path of travel, this information can be supposed to be included in the efference copy and thus be accounted for by multimodal integration, but not by our experimental measurements. Recordings were conducted on uniform terrain (e.g., a soccer field) and subjects most commonly look where they are going. Because we average over ~100–400 strides (depending on activity, see Methods), it is unlikely that a few deviant strides will have a significant impact on the mean stride cycle. However, because we estimate motor noise as the deviation from this average stride, we will interpret intentional deviations (e.g., head turns) as noise. In this way, our present estimate of motor noise can be interpreted as an upper limit on motor noise; the true motor noise is likely to be reduced relative to what we measure.

To derive our measure of sensory noise, we assume only one sensory signal, when in reality, multiple sensory signals are available for estimating head motion, in particular visual, and proprioceptive as well as vestibular signals. In order for the model to reliably predict vestibular weight these additional signals should be highly variable, such that they receive little weight and have little impact on vestibular weights predicted by the model (*w*_*vest*_ ≈ *w*_*sens*_). Alternatively, if the variability of the other sensory signals is approximately proportional to that of the vestibular signal, our estimates of vestibular weight will be smaller by a fixed constant (*w*_*vest*_ ≈ *w*_*sens*_/*c*) but still change in the same proportion over conditions. If other sensory signals are much less variable than vestibular and motor estimates, they will receive increased weight and vestibular weights will be reduced relative to model predictions. This could happen, for example, if visual self-motion signals remained reliable despite large head motions during running because of precise image stabilization via oculomotor and neck-motor reflexes. In this case, visual noise would not increase proportional to total head motion, and noise on this signal may remain small. However, to our knowledge, the amount of noise on the visual self-motion signal during running has not been quantified. Stabilization reflexes are driven predominantly by vestibular signals: a major complaint after vestibular damage is oscillopsia during walking and running, i.e., the inability to evaluate visual input due image slip caused by uncompensated head movements. Vestibular signals are known to be affected by signal-dependent noise, at least for perception (Mallery et al., 2010; Nesti et al., 2014). Thus, the visual self-motion signal will be affected not only by vestibular but also by oculomotor noise, because oculomotor signals (e.g., efference copy or proprioception) are required both for image stabilization and to convert visual motion signals from retinal to head coordinates. Overall, we suggest that the assumption of signal-dependent noise on the overall sensory self-motion estimate is fairly reasonable.

Admittedly, the strength of our conclusions is limited by the above assumptions. Nevertheless, based on these assumptions the model makes several predictions which agree with observations in the literature about difference in vestibular weighting across the stride (see next section) and across activities (see the following section). Importantly, alternative unified explanations for these observations are lacking to date.

### Model predicts differences in vestibular weight over time within a single stride

Previous studies have observed that the influence of vestibular signals on locomotor behavior varies systematically over time during the course of a single stride. For example, GVS applied either at heel contact or during toe-off, but not during the mid-stance phase (i.e., single leg support), leads to perturbed foot placement on a subsequent step (Bent et al., 2004). Similarly, when SVS is applied during locomotion, the largest correlations with EMG recorded across multiple leg muscles was observed just after heel-strike (Dakin et al., 2013).

Our model makes predictions that agree with these empirical results. It suggests that vestibular signals should be weighted most highly when *V*_*res*_ is highest (see Figure 3, bottom). Peaks in *V*_*res*_ occur just before and after the instants of peak z-axis acceleration during both walking and running. Since maximum z-axis acceleration occurs during mid-stance (Mulavara and Bloomberg, 2002), these time points correspond roughly to heel-contact and toe-off.

Phasic modulation of vestibular impact has been proposed to reflect changes in muscle activation and the impact activation will have on stabilization behavior (Dakin et al., 2013). Alternatively, it has been suggested that planning of foot placement is most active during these time points (Bent et al., 2004). Here we suggest instead that this phenomenon could reflect dynamics in relative magnitude of sensory and motor noise. Specifically, peaks in *V*_*res*_ are observed when instantaneous linear acceleration at the head is near zero, such that *SS*_*tot*_, the *V*_*res*_ denominator and the main determinant of sensory noise, is small. Note that these experiments were conducted with eyes open such that visual head motion estimates may have contributed, leading to a decrease in vestibular weight. However, unless the visual head motion estimate was very precise, the model still predicts that dynamics of vestibular and motor variability should lead to phasic modulation in vestibular weight that resembles the observed patterns.

### Model predicts differences in vestibular weight across activities

Prior research has found that vestibular perturbations have a greater impact on locomotion during walking than during running (Brandt et al., 1999; Jahn et al., 2000). This finding is interpreted as evidence that the weight given to vestibular signals varies depending on activity, in that more “automatic” behaviors, such as running, tend to rely less on sensory feedback than less automatic behaviors, such as walking slowly. Here we propose an alternative explanation for this finding, namely that the ratio of sensory to motor noise is greater for activities such as running, which leads to downweighting of sensory relative to efference copy signals.

Among prior studies that have specifically measured head motion during walking (e.g., Pozzo et al., 1990; Crane and Demer, 1997; Hirasaki et al., 1999; Mulavara and Bloomberg, 2002; Menz et al., 2003; MacDougall and Moore, 2005; Carriot et al., 2014), very few quantify spatial variability across strides. Most similar to the current approach, (Menz et al., 2003) report root-mean-square head acceleration (≈$\sqrt{{SS}_{tot}}$)as well as what they call acceleration amplitude variability (≈$\sqrt{{SS}_{res}}$) for five different walking speeds, however they do not analyze the ratio between these measures (≈$\sqrt{V_{res}}$). Judging from their data (see Figures 4, 5 in Menz et al., 2003), this ratio decreases with increasing walking speed, meaning that linear head acceleration becomes more predictable with increasing walking speed. Accordingly, our model predicts that vestibular signals should receive more weight at slower walking speed, and this agrees with prior reports demonstrating that vestibular perturbations have greater effect at slower walking speeds (Dakin et al., 2013).

In this way, the model generalizes to explain not only observed differences in vestibular reliance between running and walking (Brandt et al., 1999), but also differences as a function of walking speed (Jahn et al., 2000). These studies were conducted with blindfolded subjects, so the potential impact of visual head motion signals can be ignored. Based on the current measurements, we suggest that the model can be generalized even further and we predict that vestibular reliance is greater when walking on uneven compared to level ground (i.e., field vs. pavement), and greater for walking down than up stairs. These predictions agree with reports of vestibular patients who feel more unsteady when walking on uneven ground and when walking down stairs.

More generally, thorough testing of the model presented here would require kinematic measurements and matched measurement of vestibular weighting in the same subjects under a range of conditions, such as walking, running, walking stairs, etc. Ideally, reliance on alternative sensory cues would be reduced, e.g., by using a blindfold or otherwise reducing visual self-motion cues. Also, it is important to note that the above comparison does not consider a key difference between running and walking, which is that running does not include a double support phase during which both feet are on the ground, and often includes an aerial phase with a deterministic trajectory. Despite these qualitative differences, which likely exist for other activities as well, the model treats all activities in the same way, based on predictability of head motion.

### Physiological mechanisms for vestibulo-motor convergence and vestibular suppression

The idea that efference copies of motor commands are used in conjunction with sensory signals in order to estimate achieved movement has a long history in neuroscience (Sperry, 1950; von Holst and Mittelstaedt, 1950; Sommer and Wurtz, 2002). Several animal studies have described early integration of efference copy and vestibular sensory signals. Vestibular input for gaze stabilization during active locomotion in the tadpole (Lambert et al., 2012) and also in the adult frog (von Uckermann et al., 2013) is suppressed and substituted by spinal efference copy at the level of brainstem ocular motor neurons. Based on these results and in agreement with our data, it has been suggested that similar mechanisms hold for quadrupedal animals because of the considerable predictability of visual perturbation during locomotion as quantified by cross-correlations between head and foot movements (Chagnaud et al., 2012). Phasic modulations of vestibulospinal neurons have been documented during locomotion in quadrupeds (Matsuyama and Drew, 2000). While the relation of this modulation to muscle activations have been investigated, the relation to vestibular input has not been explored because head acceleration was not recorded simultaneously with neural activity.

For active head movements in the guinea pig, an anticipatory vestibulo-ocular reflex, driven by efference copy has been demonstrated (King, 2013). Similarly, mice and rhesus monkeys show partial suppression of vestibular input during self-generated head movements at the level of brainstem vestibular neurons (Cullen, 2014). There is evidence that the cerebellum could play a crucial role in predicting sensory consequences of movement (Cullen and Brooks, 2015). Recent findings in humans on gaze control during active head movements also underline the importance of efference copy signals in accordance with the optimal motor control framework (Saglam et al., 2014).

### Applications of the kinematic predictability metric and generalizability of the sensorimotor weighting model

Extensive prior research has investigated locomotion and gait in normal subjects as well as in elderly subjects or patients with motor or neurological deficits (e.g., Maki, 1997), and many prior studies have identified mean kinematic (e.g., stride cycle) patterns, like the ones presented here (e.g., Hirasaki et al., 1999; Mulavara and Bloomberg, 2002; Duhamel et al., 2004; Chau et al., 2005), as a way to quantify the ideal intended movement on each stride. It has been observed that stable healthy gait patterns (i.e., those associated with the lowest risk of falling) are those that exhibit the least variability (Maki, 1997; Hamacher et al., 2011), consistent with the assumption that the goal of locomotion is repeatable, deterministic motor behavior. Therefore, much attention has been devoted to methods for analyzing variability in locomotor data (Duhamel et al., 2004; Chau et al., 2005; Hamacher et al., 2011). Often, variability is quantified based on temporal characteristics such as stride timing (Hausdorff et al., 1996; Maki, 1997). Stride-to-stride stability or repeatability of spatial, kinematic measurements, such as accelerometer or positional tracking data, has been quantified multiple ways (e.g., Floquet multipliers; Dingwell and Kang, 2007, Lyapunov exponents van Schooten et al., 2011, etc, see Bruijn et al., 2009 for a comparison), but straightforward analysis of spatial variability (i.e., the KPM based on R^2^) has not been reported before. Here we show that this measure can explain degree of vestibular reliance across activities. If it can further be shown that it is a useful indicator of fall risk, it could constitute a relatively simple but useful clinical outcome measure for gait variability, not only for vestibular patients, but also for neurological or aging populations, particularly because IMU, whether mounted at the head or elsewhere on the body, constitute a low-cost and unobtrusive method for collecting clinical diagnostic data.

However, the KPM is not constrained to inertial data. Position tracking and other multiple-DOF kinematic data may be analyzed the same way. Likewise, the model of sensory and motor weight is not constrained for estimation of head motion; it applies to any situation in which achieved movement is estimated based on efference copy and a zero-mean sensory signal with signal dependent noise. In future, we expect the KPM could be applied to gain insight on sensorimotor integration for motor behaviors other than locomotion.

## Author contributions

PM and SG collaborated on all aspects of this work.

### Conflict of interest statement

The authors declare that the research was conducted in the absence of any commercial or financial relationships that could be construed as a potential conflict of interest.

## Footnotes

^1^Note that motor estimates often consider the pooled effects of efference copy and proprioceptive signals, even though proprioception is technically a sensory signal, since these are often difficult to disentangle.

^2^In the following, we no longer differentiate between genuine motor noise and variability generated by external perturbations, such as uneven ground or slipping, and subsume both under the label motor noise. The sensory effect for both motor noise and perturbations is a sensory feedback that deviates from the prediction based on the efference copy and the internal model of body and sensory properties.

## References

[B1] Bent L. R. Inglis J. T. McFadyen B. J. (2004). When is vestibular information important during walking? J. Neurophysiol. 92, 1269–1275. 10.1152/jn.01260.2003 15102904

[B2] Brandt T. Strupp M. Benson J. (1999). You are better off running than walking with acute vestibulopathy. Lancet 354:746. 10.1016/S0140-6736(99)03179-7 10475195

[B3] Bruijn S. M. van Dieen J. H. Meijer O. G. Beek P. J. (2009). Statistical precision and sensitivity of measures of dynamic gait stability. J. Neurosci. Methods 178, 327–333. 10.1016/j.jneumeth.2008.12.015 19135478

[B4] Carriot J. Jamali M. Chacron M. J. Cullen K. E. (2014). Statistics of the vestibular input experienced during natural self-motion, implications for neural processing. J. Neurosci. 34, 8347–8357. 10.1523/JNEUROSCI.0692-14.2014 24920638 PMC4051983

[B5] Chagnaud B. P. Simmers J. Straka H. (2012). Predictability of visual perturbation during locomotion, implications for corrective efference copy signaling. Biol. Cybern. 106, 669–679. 10.1007/s00422-012-0528-0 23179256

[B6] Chau T. Young S. Redekop S. (2005). Managing variability in the summary and comparison of gait data. J. Neuroeng. Rehabil. 2:22. 10.1186/1743-0003-2-22 16053523 PMC1208939

[B7] Crane B. T. Demer J. L. (1997). Human gaze stabilization during natural activities, translation, rotation, magnification, and target distance effects. J. Neurophysiol. 78, 2129–2144. 9325380 10.1152/jn.1997.78.4.2129

[B8] Cullen K. E. (2014). The neural encoding of self-generated and externally applied movement, implications for the perception of self-motion and spatial memory. Front. Integr. Neurosci. 7:108. 10.3389/fnint.2013.00108 24454282 PMC3888934

[B9] Cullen K. E. Brooks J. X. (2015). Neural correlates of sensory prediction errors in monkeys, evidence for internal models of voluntary self-motion in the cerebellum. Cerebellum 14, 31–34. 10.1007/s12311-014-0608-x 25287644 PMC4320652

[B10] Dakin C. J. Inglis J. T. Chua R. Blouin J. S. (2013). Muscle-specific modulation of vestibular reflexes with increased locomotor velocity and cadence. J. Neurophysiol. 110, 86–94. 10.1152/jn.00843.2012 23576695

[B11] Dingwell J. B. Kang H. G. (2007). Differences between local and orbital dynamic stability during human walking. J. Biomech. Eng. 129, 586–593. 10.1115/1.2746383 17655480

[B12] Duhamel A. Bourriez J. L. Devos P. Krystkowiak P. Destee A. Derambure P.. (2004). Statistical tools for clinical gait analysis. Gait Posture 20, 204–212. 10.1016/j.gaitpost.2003.09.010 15336292

[B13] Ernst M. O. Banks M. S. (2002). Humans integrate visual and haptic information in a statistically optimal fashion. Nature 415, 429–433. 10.1038/415429a 11807554

[B14] Hamacher D. Singh N. B. Van Dieen J. H. Heller M. O. Taylor W. R. (2011). Kinematic measures for assessing gait stability in elderly individuals, a systematic review. J. R. Soc. Interface 8, 1682–1698. 10.1098/rsif.2011.0416 21880615 PMC3203491

[B15] Hausdorff J. M. Purdon P. L. Peng C. K. Ladin Z. Wei J. Y. Goldberger A. L. (1996). Fractal dynamics of human gait, stability of long-range correlations in stride interval fluctuations. J. Appl. Physiol. (1985) 80, 1448–1457. 8727526 10.1152/jappl.1996.80.5.1448

[B16] Hirasaki E. Moore S. T. Raphan T. Cohen B. (1999). Effects of walking velocity on vertical head and body movements during locomotion. Exp. Brain Res. 127, 117–130. 10.1007/s002210050781 10442403

[B17] Jahn K. Strupp M. Schneider E. Dieterich M. Brandt T. (2000). Differential effects of vestibular stimulation on walking and running. Neuroreport 11, 1745–1748. 10.1097/00001756-200006050-00029 10852236

[B18] King W. M. (2013). Getting ahead of oneself, anticipation and the vestibulo-ocular reflex. Neuroscience 236, 210–219. 10.1016/j.neuroscience.2012.12.032 23370320 PMC4260774

[B19] Lambert F. M. Combes D. Simmers J. Straka H. (2012). Gaze stabilization by efference copy signaling without sensory feedback during vertebrate locomotion. Curr. Biol. 22, 1649–1658. 10.1016/j.cub.2012.07.019 22840517

[B20] MacDougall H. G. Moore S. T. (2005). Marching to the beat of the same drummer, the spontaneous tempo of human locomotion. J. Appl. Physiol. (1985) 99, 1164–1173. 10.1016/j.cub.2012.07.019 15890757

[B21] Maki B. E. (1997). Gait changes in older adults, predictors of falls or indicators of fear. J. Am. Geriatr. Soc. 45, 313–320. 10.1111/j.1532-5415.1997.tb00946.x 9063277

[B22] Mallery R. M. Olomu O. U. Uchanski R. M. Militchin V. A. Hullar T. E. (2010). Human discrimination of rotational velocities. Exp. Brain Res. 204, 11–20. 10.1007/s00221-010-2288-1 20526711 PMC2939372

[B23] Matsuyama K. Drew T. (2000). Vestibulospinal and reticulospinal neuronal activity during locomotion in the intact cat. I. Walking on a level surface. J. Neurophysiol. 84, 2237–2256. 11067969 10.1152/jn.2000.84.5.2237

[B24] Menz H. B. Lord S. R. Fitzpatrick R. C. (2003). Acceleration patterns of the head and pelvis when walking on level and irregular surfaces. Gait Posture 18, 35–46. 10.1016/S0966-6362(02)00159-5 12855299

[B25] Mulavara A. P. Bloomberg J. J. (2002). Identifying head-trunk and lower limb contributions to gaze stabilization during locomotion. J. Vestib. Res. 12, 255–269. 14501102

[B26] Nesti A. Barnett-Cowan M. Macneilage P. R. Bulthoff H. H. (2014). Human sensitivity to vertical self-motion. Exp. Brain Res. 232, 303–314. 10.1007/s00221-013-3741-8 24158607 PMC3898153

[B27] Pozzo T. Berthoz A. Lefort L. (1990). Head stabilization during various locomotor tasks in humans. I. Normal subjects. Exp. Brain Res. 82, 97–106. 10.1007/BF00230842 2257917

[B28] Roy J. E. Cullen K. E. (2004). Dissociating self-generated from passively applied head motion, neural mechanisms in the vestibular nuclei. J. Neurosci. 24, 2102–2111. 10.1523/JNEUROSCI.3988-03.2004 14999061 PMC6730417

[B29] Saglam M. Glasauer S. Lehnen N. (2014). Vestibular and cerebellar contribution to gaze optimality. Brain 137, 1080–1094. 10.1093/brain/awu006 24549962

[B30] Sommer M. A. Wurtz R. H. (2002). A pathway in primate brain for internal monitoring of movements. Science 296, 1480–1482. 10.1126/science.1069590 12029137

[B31] Sperry R. W. (1950). Neural basis of the spontaneous optokinetic response produced by visual inversion. J. Comp. Physiol. Psychol. 43, 482–489. 10.1037/h0055479 14794830

[B32] van Beers R. J. Sittig A. C. Gon J. J. (1999). Integration of proprioceptive and visual position-information, An experimentally supported model. J. Neurophysiol. 81, 1355–1364. 10085361 10.1152/jn.1999.81.3.1355

[B33] van Schooten K. S. Sloot L. H. Bruijn S. M. Kingma H. Meijer O. G. Pijnappels M.. (2011). Sensitivity of trunk variability and stability measures to balance impairments induced by galvanic vestibular stimulation during gait. Gait Posture 33, 656–660. 10.1016/j.gaitpost.2011.02.017 21435878

[B34] von Holst E. Mittelstaedt H. (1950). Das Reafferenzprinzip-(Wechselwirkungen Zwischen Zentralnervensystem Und Peripherie). Naturwissenschaften 37, 464–476. 10.1007/BF00622503

[B35] von Uckermann G. Le Ray D. Combes D. Straka H. Simmers J. (2013). Spinal efference copy signaling and gaze stabilization during locomotion in juvenile *Xenopus frogs*. J. Neurosci. 33, 4253–4264. 10.1523/JNEUROSCI.4521-12.2013 23467343 PMC6704964

